# Puf3p induces translational repression of genes linked to oxidative stress

**DOI:** 10.1093/nar/gkt948

**Published:** 2013-10-25

**Authors:** William Rowe, Christopher J. Kershaw, Lydia M. Castelli, Joseph L. Costello, Mark P. Ashe, Christopher M. Grant, Paul F. G. Sims, Graham D. Pavitt, Simon J. Hubbard

**Affiliations:** ^1^The Faculty of Life Sciences, The Michael Smith Building, The University of Manchester, Oxford Road, Manchester M13 9PT, UK and ^2^Manchester Institute of Biotechnology (MIB), Faculty of Life Sciences, The University of Manchester, 131 Princess Street, Manchester M1 7DN, UK

## Abstract

In response to stress, the translation of many mRNAs in yeast can change in a fashion discordant with the general repression of translation. Here, we use machine learning to mine the properties of these mRNAs to determine specific translation control signals. We find a strong association between transcripts acutely translationally repressed under oxidative stress and those associated with the RNA-binding protein Puf3p, a known regulator of cellular mRNAs encoding proteins targeted to mitochondria. Under oxidative stress, a *PUF3* deleted strain exhibits more robust growth than wild-type cells and the shift in translation from polysomes to monosomes is attenuated, suggesting *puf3Δ* cells perceive less stress. In agreement, the ratio of reduced:oxidized glutathione, a major antioxidant and indicator of cellular redox state, is increased in unstressed *puf3Δ* cells but remains lower under stress. In untreated conditions, Puf3p migrates with polysomes rather than ribosome-free fractions, but this is lost under stress. Finally, reverse transcriptase-polymerase chain reaction (RT-PCR) of Puf3p targets following affinity purification shows Puf3p-mRNA associations are maintained or increased under oxidative stress. Collectively, these results point to Puf3p acting as a translational repressor in a manner exceeding the global translational response, possibly by temporarily limiting synthesis of new mitochondrial proteins as cells adapt to the stress.

## INTRODUCTION

Cells maintain homeostasis by eliciting a variety of responses to environmental stress. In the postgenomic era, it is common to measure these responses at the transcriptional level using microarrays or more recently RNA-seq (1). These data are independent of rates of protein synthesis and degradation and as such often poorly reflect changes at the proteomic, metabolic or systems level where additional levels of control are known to act (2). Ideally, these effects should be measured or modelled to properly understand how gene function is regulated under stress, particularly at the translational level. For example, bioinformatics approaches have attempted to reconcile some of these differences and account for translational control, through the generation of explicit rules based on the sequence features associated with the open reading frame (ORF) (3–5). One such example is the translational adaptation index (tAI), which correlates codon usage with abundance of tRNAs (5), recently updated to reflect codon demand as well as supply (6). Such rules are not necessarily comprehensive for complete gene sets and may extrapolate poorly to stress conditions.

Translation is regulated at the initiation phase (7,8), where the small ribosomal (40S) subunit is recruited to the 5′ end of the mRNA with the aid of the eukaryotic initiation factors (eIFs) and scans the leader sequence to locate the start codon. Under differing stress conditions, initiation can be inhibited by interference with various steps in the eukaryotic initiation process, leading to a cessation of translation. For instance the mRNA cap-binding protein eIF4E can be prevented from recruiting the 40S ribosomal subunit to the cap structure by competitive binding of inhibitory proteins (9). Additionally, eIF2, the initiation factor responsible for binding Met-tRNA^i^ to the ribosome, can be inhibited by specific eIF2α kinases (10) to exert a programmed response to a variety of stresses including amino acid starvation. This ‘global’ response to translational regulation is not applied evenly across all mRNAs. One well-characterized example from yeast, amino acid starvation, leads to eIF2α phosphorylation by the eIF2α kinase Gcn2p down-regulating overall translational initiation. In contrast, *GCN4* encoding the transcription factor Gcn4p is translationally activated in response to amino acid starvation. Translational control is mediated by altered translation of four upstream open reading frames (uORFs) in the mRNA sequence (11). Elevated Gcn4p levels facilitate enhanced synthesis of amino acid biosynthesic enzymes thereby ameliorating the imposed stress. Global studies of translational control in response to amino acid starvation have revealed that the translation of hundreds of mRNAs are maintained or enhanced following amino acid starvation (12,13). Many of these do not possess uORFs, indicating that other mRNA-specific controls operate.

The elements responsible for specific mRNA control of translation are expected to be encoded by sequences and/or structures within individual mRNAs. The effects of structural features such as IRES elements (14), pseudoknots (15), microRNAs (16), uORFs (11) and specific protein–RNA interactions (17) can combine to orchestrate the necessary regulation of protein synthesis. In *Saccharomyces cerevisiae*, a recent survey revealed over 600 potential RNA-binding proteins (17). While much effort has been made to unravel the co-ordination of gene expression by transcription factors (18), the corresponding control of mRNA fate by these posttranscriptional RNA-binding proteins has received less attention. To understand the regulatory nature of mRNA-binding proteins, it is essential to determine their mRNA targets. Hogan *et al.* (17) systematically investigated the binding partners of 40 mRNA-binding proteins in *S. cerevisiae,* revealing that >70% of the yeast transcriptome is associated with at least one of these proteins and on average each mRNA associates with three proteins (17). This landmark study, and others (19–23), have clarified the widespread role for RNA-binding proteins in regulation of gene expression at the posttranscriptional level. They demonstrate that more effort is required both to characterize the functional significance of these events, and to map the binding partners of hundreds more RNA-binding proteins. The ultimate goal is to determine their role in what appear to be complex networks of posttranscriptional control.

Commonly, the extent of translational control in a system is measured through a technique known as polysome profile analysis (24). Once a ribosome has begun translational elongation, other ribosomes can initiate translation on the same mRNA; it is common for as many as 10 ribosomes to be translating the same mRNA at any one time (24), with some dependency on transcript length. These actively translated, polysomally associated mRNAs can be easily separated from less active mRNAs using a sucrose density gradient and the distribution of specific mRNAs can be quantified via microarray analysis or sequencing. This approach has been used to determine the translational status of mRNAs in response to a variety of stimuli (13,18,25–30). We studied the properties of transcripts that are translationally up-regulated and down-regulated in response to various stress conditions: low peroxide, high peroxide (30), amino acid starvation (13), butanol addition (18) and glucose starvation (31) in addition to knockout of proteins implicated in translational regulation (25). We class ‘up’ and ‘down’ regulation as relative translational states that indicate mRNAs that behave significantly different from the majority of genes in microarray analyses performed on polysomal fractionated cell extracts. Since the general trend under each applied stress is to reduce translation initiation, ‘up-regulation’ refers to genes whose polysome distribution is maintained or increased following stress, while ‘down-regulation’ refers to those mRNAs whose polysome distribution is dramatically reduced following stress. For simplicity, we refer to up- and down-regulation throughout this manuscript.

To attempt to explain how translational regulation may be exerted on specific mRNAs, we used a simple comparison of means via *t*-tests to identify a potential role for 5′UTRs and uORFs in translational control (32). More recent studies have continued this work, highlighting similar sequence and structural enrichments within translationally up-regulated gene data sets (33). Although both these (and other) studies point to the roles of various genetic features, including uORFs, 5′UTR length and structure, presence/absence of motifs, it is clear that the signals exploited for translational control are multifactorial and likely interdependent. For example, only 13% of yeast transcripts are expected to have uORFs in their 5′ UTRs (32) and it is clear that multiple 5′ transcriptional start sites and poly-A tails are used (1,32,34,35). Simple statistical techniques are unable to capture the complexity that is required to co-ordinate translation as they are incapable of identifying these complex interactions between sequence and structural features within the mRNAs.

In this study, we expand on previous work by combining 65 features describing each mRNA sequence and use them to train a non-linear classifier; a Random Forest (36), to predict the sequence properties that determine translational control under oxidative stress conditions. A classifier is produced with strong predictive accuracy (as tested by cross validation); however, this is not the sole aim of the approach. By identifying motifs for specific RNA-binding proteins within the transcripts, we highlight the strong association of Puf3p affinity with the translationally down-regulated peroxide data set. Puf3p is an RNA-binding protein from the PUmilio-Fem-3-binding factor (PUF) family known to promote RNA degradation and facilitate trafficking of mRNA to the mitochondrion (37,38). We have experimentally tested the role of Puf3p in the translational response to oxidative stress. We find that both growth and translation of a *puf3Δ* strain are significantly more resistant to hydrogen peroxide stress, while the growth of knockout strains for other PUF genes are still as sensitive as wild type. Consistent with this, the *puf3Δ* strain exhibits an increased ratio of reduced to oxidized glutathione, a major cellular antioxidant and a higher reduced:oxidized ratio is maintained following H_2_O_2_ addition. Puf3p migrates with polysomes in unstressed cells, an interaction that is lost following stress where a significant fraction shifts to the sub-polysomal fractions. Puf3-TAP immunoprecipitation in combination with quantitative reverse transcriptase-polymerase chain reaction (qRT-PCR) indicates that Puf3p remains bound to its target mRNAs after oxidative stress. Collectively, these data highlight a novel function for Puf3p in the repression of translation and/or induced mRNA decay of specific mRNAs in *S. cerevisiae* in response to oxidative stress. A large fraction of Puf3p mRNA targets encode proteins targeted to mitochondria, in line with its role in oxidative stress (39). We suggest that our findings represent part of an appropriate physiological response mechanism to down-regulate expression of proteins targeted to mitochondria in response to the perceived damaging environment.

## MATERIALS AND METHODS

### Microarray analysis

The original transcription profiling and translation status under oxidative stress data sets were downloaded from ArrayExpress (accession number E-MEXP-526) in the processed form [details of initial array normalization are given in (30)]. The data sets include the log_2_ intensity values for total, monosome and polysome associated transcripts in the presence and absence of 0.2 mM H_2_0_2_ (low peroxide) and 2 mM H_2_0_2_ (high peroxide). The translation state for each mRNA was determined by calculating the log_2_ ratio of intensities in the polysome compared with the monosome fractions for both control and stress conditions [log_2_(polysome_control_:monosome_control_) and log_2_(polysome_stress_:monosome_stress_)]. A second ratio of these two values gives the change in translation state [log_2_(polysome_control_:monosome_control_):log_2_(polysome_stress_:monosome_stress_)]. Transcripts were defined as being translationally up-regulated or down-regulated if the change in translation state was >1.0 (a 2-fold change in absolute ratios). To ensure the statistical significance of these transcripts, a two-factor ANOVA model using Limma (40) functions *lmFit* and *eBayes* was applied to the two ratios. Significant genes were selected with interaction adjusted *P*-values of *P* < 0.01.

### Training and testing the random forest

Random forests are an ensemble classifier with high accuracy, based on the output from many classification/regression trees (36), which have been used in a variety of applications including the analysis of sequence data (41), genome-wide association studies (42) and quantitative proteomics (43,44). They accept both discrete and continuous features as input data and provide two standard methods to measure feature importance when constructing the constituent trees. The first is based on permuting the predictor variables and then calculating the error rate for classification using the out-of-bag data. The second is the Gini index (a measure of node impurity) for each feature. The sum of all the Gini indices for each tree in the Random Forest gives a measure of importance comparable with that measured by permutation.

Training and testing of the input data was performed with the RandomForest package in R (36). Performance of the Random Forest at predicting translationally up-regulated and down-regulated mRNAs was assessed in two ways. Firstly, a confusion matrix was generated based on the Out Of Box (OOB) error estimate (45). This involved constructing trees within a Random Forest using bootstrapping of the original sample, omitting around one-third of the original data, and subsequently using these remaining data to obtain a running estimate of classification error. Secondly, performance was measured by generating a receiver operator characteristic (ROC) curve using the ‘performance’ class from the ROCR R package (46). ROC curves measure the performance of a binary classifier in terms of number of true positives and numbers of false positives in response to variation in the discrimination threshold. The area under the ROC curve gives a unary measure of performance between zero and one, where 0.5 is random and 1.0 is perfect. The ROC curve was derived from data generated from 10-fold cross-validation.

When generating the training set each transcript was subdivided into three sections: 5′UTR, 3′UTR and ORF [5′ and 3′ UTR ends were defined using the experimentally measured data set generated in (47), excluding mRNAs without defined 5′ or 3′UTRs from the analysis]. Features were then generated that described these regions, composed of base composition, structural free energy (48) and binding sites for RNA-binding proteins (49). More general features describing the ORF were also included: codon adaptation index (CAI) (3), frequency of optimal codons (fop) (50), tAI (5) and amino acid composition of the translated protein; in addition AUG-CAI(r) (51), presence of start and stop codons and GC content before and after the start codon [features previously identified as important in translational control (52)] were also included. In total, 77 features were generated for each mRNA under consideration. Further details on how the training set was generated are given in Supplementary Table S1.

Binary classification was made in a pairwise fashion between translationally activated transcripts and all other transcripts in the gene set, and between translationally down-regulated transcripts and the other transcripts within the gene set. The two parameters ‘ntree’ (number of trees in the classification) and ‘mtry’ (the number of features tried at each node) were tuned to yield the lowest OOB classification error. The classes were weighted to match the proportion of observations in each class.

### GO-term enrichment

The Gene Ontology GO-Slim mapper tool from SGD (http://www.yeastgenome.org) was used to identify GO-slim terms associated with the transcripts of interest. Enrichments were calculated using hypergeometric tests and corrected for multiple testing using the method described by Benjamini and Hochberg (53). Terms associated with multiple groups of transcripts were clustered (using average linkage hierarchical clustering) and visualized with TreeView (54).

### Knockout strain growth and Glutathione analysis

The yeast strain BY4741 (*MAT***a** *his3*Δ1 *leu2*Δ0 *met15*Δ0 *ura3*Δ0) (Euroscarf) and the *puf1::kanMX4*, *puf2::kanMX4*, *puf3::kanMX4*, *puf4::kanMX4*, *puf5::kanMX4* and *puf6::kanMX4* strains (Euroscarf) in the same background were grown in rich YEPD medium (2%, wt/vol, glucose; 2%, wt/vol, bactopeptone; and 1%, wt/vol, yeast extract) (55) at 30°C. Media were solidified by the addition of 2% (wt/vol) agar. Stress sensitivity was determined by growing cells to stationary phase and spotting a serial dilution of each strain onto agar plates containing 14 mM hydrogen peroxide. Reduced (GSH) and oxidized glutathione (GSSG) levels were determined as described previously (56).

### Quantitative RT-PCR

Quantitative RT-PCR was performed on samples collected from total and immunoprecipitated RNA samples under stress and control conditions. Puf3p-TAP yeast (*MAT***a** *his3*Δ1 *leu2*Δ0 *met15*Δ0 *ura3*Δ0 *PUF3*::TAP-HIS3) (Open Biosystems) were grown in SCD-His medium (Formedium) to A_600_ = 0.6 in parallel cultures and one-half was treated with 0.4 mM H_2_O_2_ for 15 min before cell harvest by centrifugation. Cell pellets were rapidly frozen in liquid nitrogen to maintain their translation state and subsequently lysed into Buffer A (20 mM Tris–HCl, pH 8, 140 mM NaCl, 1 mM MgCl_2_, 0.5% NP40, 0.5 mM DTT, 1 mM PMSF, EDTA free Protease Inhibitor cocktail tablet (Roche), 100 µM NaV_3_O_4_, 5 mM NaF (phosphatase inhibitors) and 40 U/ml RNAsin) using liquid nitrogen and a 6870 Freezer Mill (Spex). Cell lysates were cleared by centrifuging twice at 15 000*g*. Five percent of the sample was reserved for isolation of total RNA isolation (see below). The remaining fraction was used for TAP affinity purification of Puf3p-TAP associated RNA using Tosyl-activated Dynabeads M-280 magnetic beads (Dynal).

Coupling of Rabbit IgG to Tosyl-activated Dynabeads M-280 magnetic beads was performed as follows: 10-mg beads were resuspended and washed twice with 0.1 M Na-phosphate buffer (pH 7.4). Next, 975 μl of a solution of 62.8 mM Na-phosphate, pH 7.4, 0.38 mg/ml IgG and 1 M ammonium sulphate, pH 7.4, was added. Beads were incubated for 20 h at 37°C with gentle agitation to bind IgG. After conjugation, beads were washed with 1 ml phosphate buffered saline (PBS), then 1 ml 200 mM Glycine–HCl (pH 2.5) to remove excess IgG and then 1 ml 100 mM Tris, pH 8, to neutralize. Next, 1 ml 100 mM Triethylamine wash followed by a 1-ml 100 mM Tris, pH 8, to neutralize. Next a 1-ml PBS + 0.5% bovine serum albumin (BSA) wash was followed with a 1-ml 100 mM Tris, pH 7.4, 0.5% BSA wash to block and deactivate any remaining reactive groups. A 1-ml PBS + 0.1% BSA + 0.5% NP40 wash was used to remove physically adsorbed but not covalently attached proteins. Finally the washed beads were resuspended in 200 μl PBS + 0.1% BSA + 0.02% Sodium Azide storage buffer at 4°C.

For TAP affinity purification, 10-mg beads were pre-washed three times with Buffer A and then added to 4 mg/ml of lysate. Immunoprecipitations were performed for 20 min at 4°C and washed five times with Buffer A containing 10 U/ml RNAsin, changing tubes at least twice during the washes and the final two washes were performed for 15 min each. For RNA isolation after the final wash, the beads were resuspended in 250 µl of Buffer A and treated with Trizol (Ambion). The aqueous phase was mixed with 70% ethanol and the RNA precipitated. RNA was converted to cDNA using a Protoscript M-MuLV *Taq* RT-PCR kit (New England biolabs). Oligonucleotide primers (Supplementary Table S2) were designed to specific transcripts that are either differentially regulated translationally under conditions of oxidative stress, were identified as associating with Puf3p (17,57), are mitochondrially associated as classified by SGD (58) or a combination of these factors.

Quantification was performed using the CFx Connect Real-Time system with iTaq Universal SYBR Green Supermix (BioRad Laboratories). Samples were run in triplicate and in each instance signals were normalized to the untreated input for each primer pair used.

### Immunoblotting

Cell extracts, polysome fractionation and protein preparation were performed as described previously (31). Cultures (SCD-His grown to A_600_ 0.6–0.7) were treated with 0.4 mM hydrogen peroxide for 15 min, where indicated, before cell harvest, lysis and polysome analysis on 15–50% sucrose gradients. Where ribosomes were dissociated into 40S and 60S subunits, polysome extracts were treated with 20 mM EDTA for 10 min on ice before being resolved on a 5–25% sucrose gradient. Fractions were collected and analysed by western blotting. Puf3p-TAP was detected by PAP antibody (Sigma); all other proteins were detected by antibodies raised against the endogenous proteins.

## RESULTS

**Figure 1.**
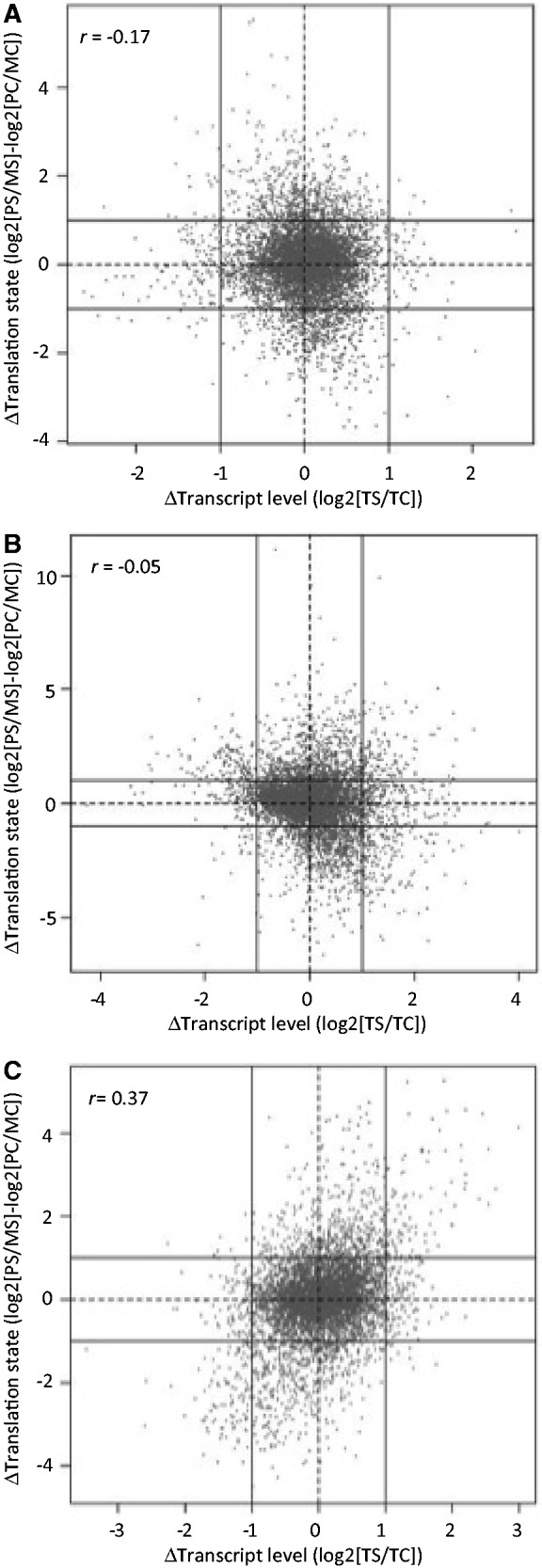
Absence of concerted potentiation in yeast gene expression under peroxide stress. The correlation between transcriptional and translational response under (**A**) low peroxide, (**B**) high peroxide, (**C**) amino acid deprivation stress conditions (13,30). Individual points represent changes in gene expression as measured by array, where MC = monosomal control, MS = monosomal stress, PC = polysomal control, PS = polysomal stress, TC = transcript control, TS = transcript stress. Solid lines are added to the plots to indicate a 2-fold change.

**Figure 2.**
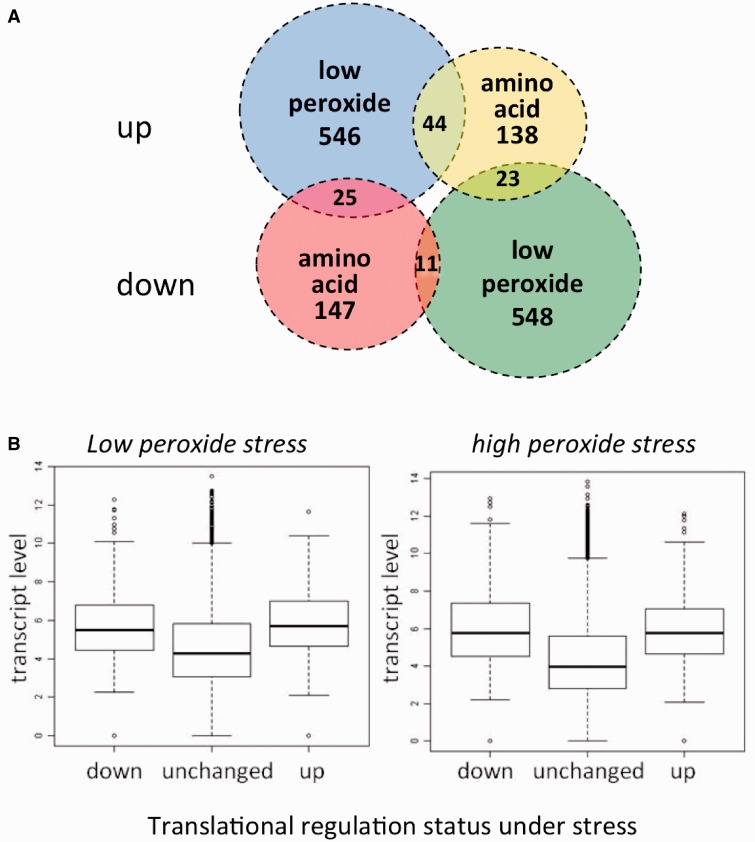
Properties of genes displaying differential translational regulation under stress. (**A**) Euler diagram displaying the co-occurrence of transcripts in the translationally up- and down-regulated data sets under low-peroxide and amino acid stress conditions. Gene sets were generated from a reanalysis of previously published microarray data sets (13,30) (see ‘Materials and Methods’ section). (**B**) Association between mRNA levels and translationally regulated genes under peroxide stress. Boxplots displaying transcript levels (after stress) of genes identified as being differentially polysomally associated (up), repressed (down) or unchanged under low peroxide and high peroxide stress conditions.

Under the influence of many stress conditions, e.g. heat shock and amino acid depletion, changes at the transcriptional level can be integrated with changes in the translational state in an effect known as ‘potentiation’ (59). It has been previously reported that little or no such co-ordination is observed between changes in transcription and translation under oxidative stress in *S. cerevisiae*, and this is highlighted in Figure 1, where there is, if anything, a negative correlation observed between transcriptional and translational change. Regardless of this lack of synchronicity under oxidative stress, there is a strong enrichment of GO-terms associated with the translationally regulated mRNAs (both up and down), indicating a co-ordinated underlying control mechanism (30). This lack of correlation is in contrast to that observed under amino acid depletion where both transcription and translation responses appear, to a large degree, co-ordinated with a modest Pearson correlation of *r* = 0.37 (Figure 1C). Both amino acid and oxidative stress responses activate Gcn2p causing phosphorylation of eIF2α and subsequent global translational repression as well as activation of *GCN4* translation. However, the majority of genes translationally regulated differ between these stresses (Figure 2A), suggesting strongly that other ‘stress-specific’ factors contribute to each response (13,30).

From our work and others, the peroxide response stands out among translatome data sets due to the high number of translationally activated and deactivated transcripts under stress (>500 both activated and deactivated under the two peroxide conditions). These transcripts have a low overlap with mRNAs translationally controlled in other stress data sets (see Figure 2A for comparison with amino acid starvation) indicating a distinct underlying mechanism of response. We also note that, in general, there is not a single ‘core response’ set of genes in *S. cerevisiae* that are translationally activated in response to stress as has been observed in fission yeast (60). For a robust machine learning analysis to be feasible, we require a large number of data points in each of the classes, making the peroxide translatome data sets of particular interest and best placed for investigation.

### Random forest classification reveals Puf3p target mRNAs are translationally repressed following oxidative stress

The Random Forest produces a powerful classifier capable of distinguishing translationally up-regulated and down-regulated transcripts from the total gene set. It is evident from the confusion matrix generated by the OOB data set and the ROC analysis generated through 10-fold cross-validation, that the predictive accuracy as measured by the area under the ROC curve is high (between 0.74 and 0.85; see Table 1). This is despite the inherent noise that might be expected when determining the translational status of each of the transcripts (both experimentally, and via the likely propagation of error when working with the ratio of ratios used as a metric for translational status). We note that superior performance is obtained from the classifiers trained using data for the low peroxide concentration. This is consistent with our expectation that the higher concentration is in part damaging to the cells, i.e. affects many biological processes including other steps in the translation process (30).

**Table 1.** Confusion matrix based on random forest classification of translationally up- and down-regulated transcripts under peroxide stress

Condition	Response	TP	FP	TN	FN	AUC	Top3 important features	Effect
Low peroxide	Down	387	64	2408	1357	0.84	Puf3 site in 3′UTR	+
							Length of ORF	−
							Serine in translated ORF	−
	Up	392	72	2592	1160	0.85	Transcription level (stress)	+
							Transcription level (control)	+
							%Adenine in ORF	−
High peroxide	Down	647	173	2041	1355	0.78	Puf3 site in 3′UTR	+
							CAI	−
							Length of ORF	−
	Up	530	145	2045	1496	0.74	Transcription level (control)	+
							Transcription level (stress)	+
							%Adenine in ORF	−

Area under the ROC curve (AUC) (see ‘Materials and Methods’ section) and important features (based on the Gini index) are also shown (TP, true positives; FP, false positives; TN, true negatives; FN, negatives in the classification). Effect determined by positive (+) or negative (−) correlation.

The top three features corresponding to each of the translational responses as determined by the Gini index, which scores the utility of the feature in distinguishing the gene sets, are shown in Table 1 (full rankings of all features are given in Supplementary Table S1). In all instances, the underlying significance provided by the total transcript levels on the classification is high, most notably for the up-regulated gene sets. This is despite the absence of correlation observed between transcription and translation status in Figure 1 (*r* = −0.17, *r*^2 ^= 0.03 for low peroxide and *r* = −0.05, *r*^2 ^= 0.0025 for high peroxide, *r* = 0.37, *r*^2 ^= 0.1 for amino acid depletion, all correlations are significantly different to a correlation of 0, with *P* <2.2 × 10^−^^16^). This indicates the effect is not explained by, or a consequence of, potentiation. Figure 2B shows the distributions of the transcript levels of different mRNA sets classified by their translational state (induced, repressed or unchanged) under oxidative stress conditions. When comparing unchanged transcripts to those that exhibit a large translational response, a Wilcoxon rank sum test between induced (up) versus unchanged, and repressed (down) versus unchanged reveals a strong statistical difference (*P* < 2.2 × 10^−^^16^ in each test). This highlights the deviation between the cellular transcript ‘level’ and translational ‘response’ to stress and may indicate a necessity to fine-tune control of expression of these abundant transcripts via control of translation.

The second feature that stands out in the classification is the importance of the Puf3p motif in the 3′UTRs of transcripts within the peroxide stress down-regulated data set. The PUF proteins are a highly conserved family of proteins related to the Pumilio protein of *Drosophila melanogaster* and Fem-3-binding factor of *Caenorhabditis elegans* (61). In yeast there are six PUF proteins, that are known to bind conserved sequence motifs predominantly in the 3′UTRs of groups of functionally related mRNAs and mediate gene expression through control of translation (62) and/or transcript degradation (63). The family of proteins are characterized by a series of eight repeats of the Pumilio-Homology domain, which fold to form a repeated three-helix domain that confers RNA binding (64). Puf3p is known for its high specificity for transcripts of nuclear encoded proteins that localize to the mitochondrion. So strong is the association that the characteristic binding site (CYUGUAAAUA) was discovered in these functionally related transcripts before the identification of the associated protein (65). Among the PUF proteins in *S. cerevisiae*, Puf3p shares the greatest similarity to the human homologue PUM; however, the prevalence for PUM binding exclusively to nuclear encoded mitochondrial proteins is not conserved (66). Puf3p localizes to the cytoplasmic outer surface of mitochondria and is known to play roles in mitochondrial mobility (37) and the control of mRNA deadenylation and degradation (38). A recent study by Chatney-Lapointe and Shardel (67) also indicated a role for Puf3p in the control of translation of nuclear encoded mitochondrial transcripts. Notably, oxidative stress is also known to be tightly coupled to mitochondrial function, as the primary source of reactive oxygen species (ROS) in the eukaryotic cell via oxidative phosphorylation (68).

**Figure 3.**
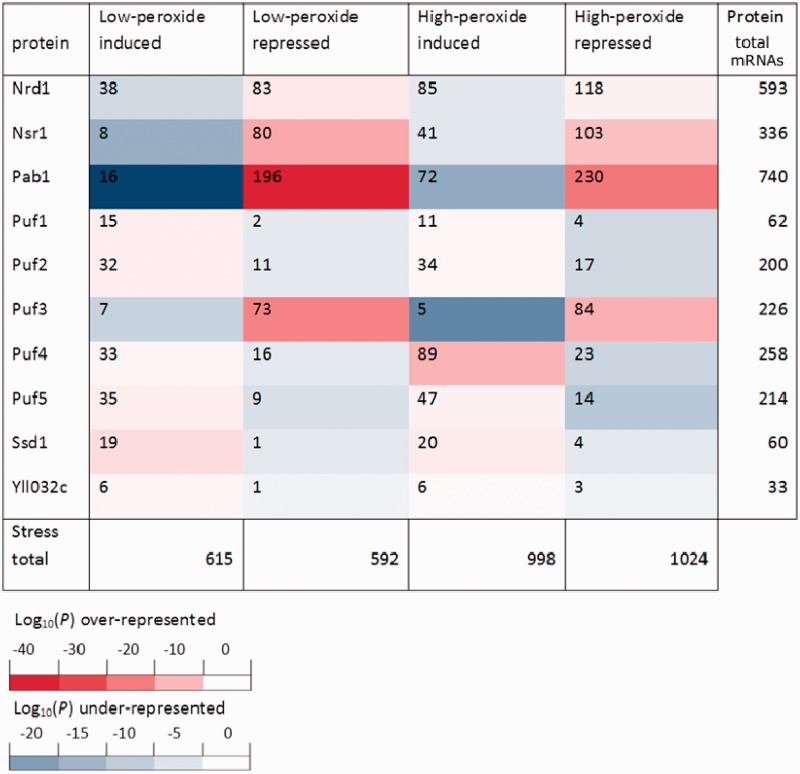
RNA-binding protein enrichments in peroxide stress data sets. Displaying the co-association of transcripts identified as being co-purified with different RNA-binding proteins with motifs reported by Riordan *et al.* (plus Puf1) (14,43) and those translationally activated/repressed under oxidative stress (lp = low-peroxide, hp = high-peroxide) conditions. The table is coloured according to the *P*-value of this co-association based on a hyper-geometric test (corrected for multiple testing using Benjamini and Hochberg method).

**Figure 4.**
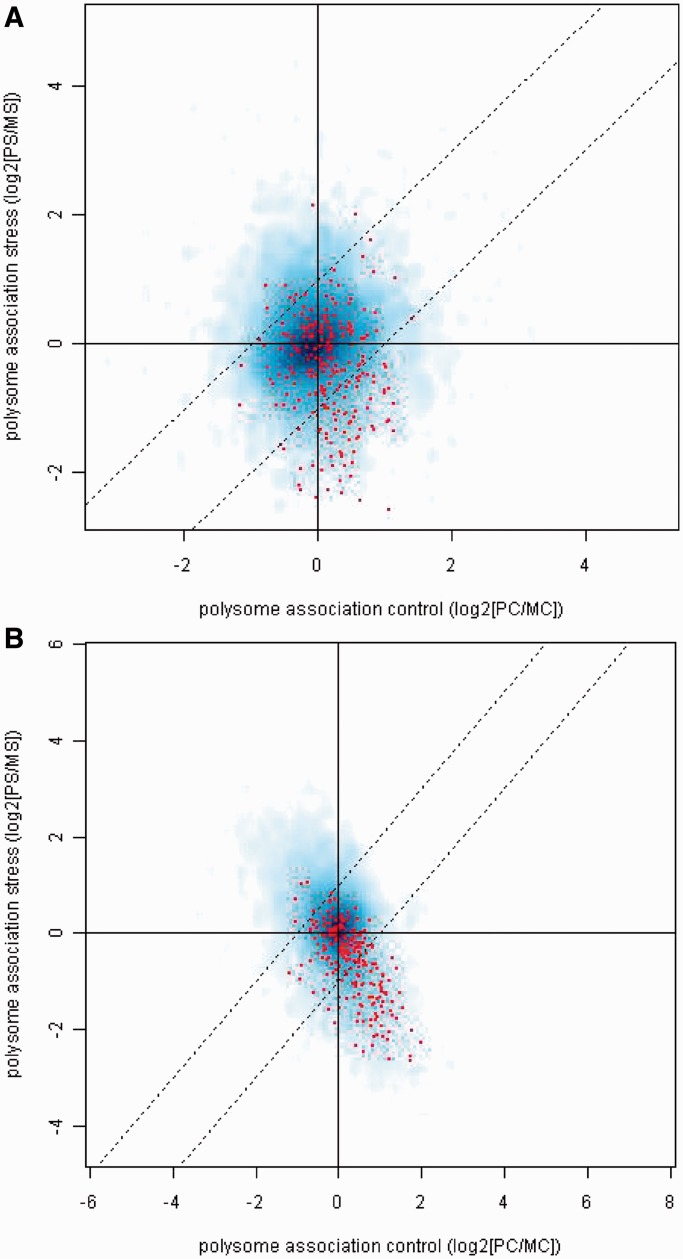
Puf3p associates with translationally repressed transcripts under oxidative stress. Smooth scatterplots relating levels of translation under control conditions and (**a**) low peroxide stress (**b**) high peroxide stress, with transcripts co-purified with Puf3p highlighted in red [log_2_(polysome_control_:monosome_control__)_ versus log_2_(polysome_control_: monosome_control_)].

Notably, the mRNA targets for five of the six PUF proteins have been characterized in *S. cerevisiae* (49). Here, for the first time, we place this data in the context of stress-dependent translational control data sets. Figure 3 displays the coincidence of transcripts significantly associated with the various PUF proteins, and those translationally down-regulated or up-regulated during oxidative stress. The strongest co-association with PUF proteins is between the mRNAs that are translationally down-regulated under low peroxide conditions and those transcripts with strong affinity for Puf3p. Clearly, there is a relationship between the Puf3p targets and the peroxide stress data sets, with transcripts enriched in the up-regulated peroxide set under-represented in the Puf3p targets and vice versa. The distribution of the Puf3p-binding transcripts in terms of translational response in the two peroxide stress data sets is shown in Figure 4 (red dots), displaying a shift below the midline towards translational inhibition. None of the other PUF protein data sets fully replicate this trend. Two other known RNA-binding proteins from Figure 3 display similar behaviour; Pab1p and Nsr1p. Notably, both these proteins have previously been shown to have a high co-association with the same transcripts that Puf3p binds (69). In particular, Pab1p is the most enriched protein co-associated with transcripts pulled down with Puf3p in an RNA-dependent fashion (23). These proteins were not identified in the Random Forest. We attribute this to the fact that motif scores were used in the machine learning model, as opposed to presence/absence from protein pull-down experiments, and the consequent discrepancies between these two approaches to characterize RNA binding protein (RBP) targets. The less-specific nature of Pab1p binding, which recognizes the poly-A tails ubiquitous to all mature mRNAs, also means that it is harder to characterize through motif analysis. Consequently, it is much less likely to be identified through the Random Forest analysis. Although Nsr1p has a characterized motif, it is less specific than Puf3p’s and, as noted above, they apparently share many targets. It has previously been noted that mRNAs that interact with Puf3p are transcriptionally up-regulated in response to oxidative stress (69), which would be consistent with the increased mRNA levels observed in translationally repressed transcripts following oxidative stress (see Figure 2B).

**Figure 5.**
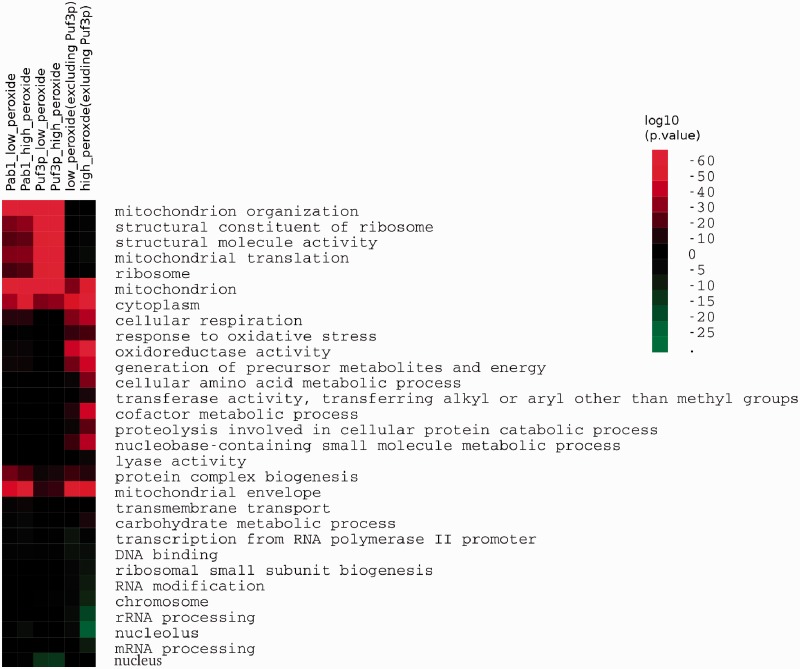
GO-term association of selected gene sets. Over-representation of biological process GO-terms associated with transcripts down-regulated in low/high peroxide stress conditions, which are bound to puf3 and pab1. Puf3 and Pab1 binders are excluded from columns 5 and 6 as a control. The key shows the significance, expessed as a log_10_ *P*-value, of the term enrichment in the different data sets.

Figure 5 displays the over-represented GO-terms associated with the transcripts found to be both associated with Puf3p and Pab1p and down-regulated during peroxide stress. The high overlap between the GO-terms highlights the consistency in the two sets of targets. Because of Puf3p’s known association with the mitochondrion (37,70,71), it is encouraging that these transcripts are highly enriched with terms relating to mitochondrial activity. Similarly it has previously been reported that Puf3p preferentially interacts with mRNAs of proteins that form structural constituents of the ribosome (61), which is also matched by our independent results. As a control we also examined the GO-term enrichment of those transcripts that were translationally down-regulated in response to oxidative stress but which lack Puf3p-binding sites. In these data, we again find enrichment in the transcripts of proteins located in the mitochondrion and general stress response, but not in specific categories linked to mitochondrial translation at the top of the heatmap. Puf3p is known to interact with the mitochondrial translational machinery (71) and this further highlights Puf3p’s likely causal role in the regulation of translation in response to oxidative stress. This suggests this is mediated via the binding of Puf3p to elements in the transcripts of these mitochondrially active proteins.

### Puf3p loses association with polysomes following oxidative stress

**Figure 6.**
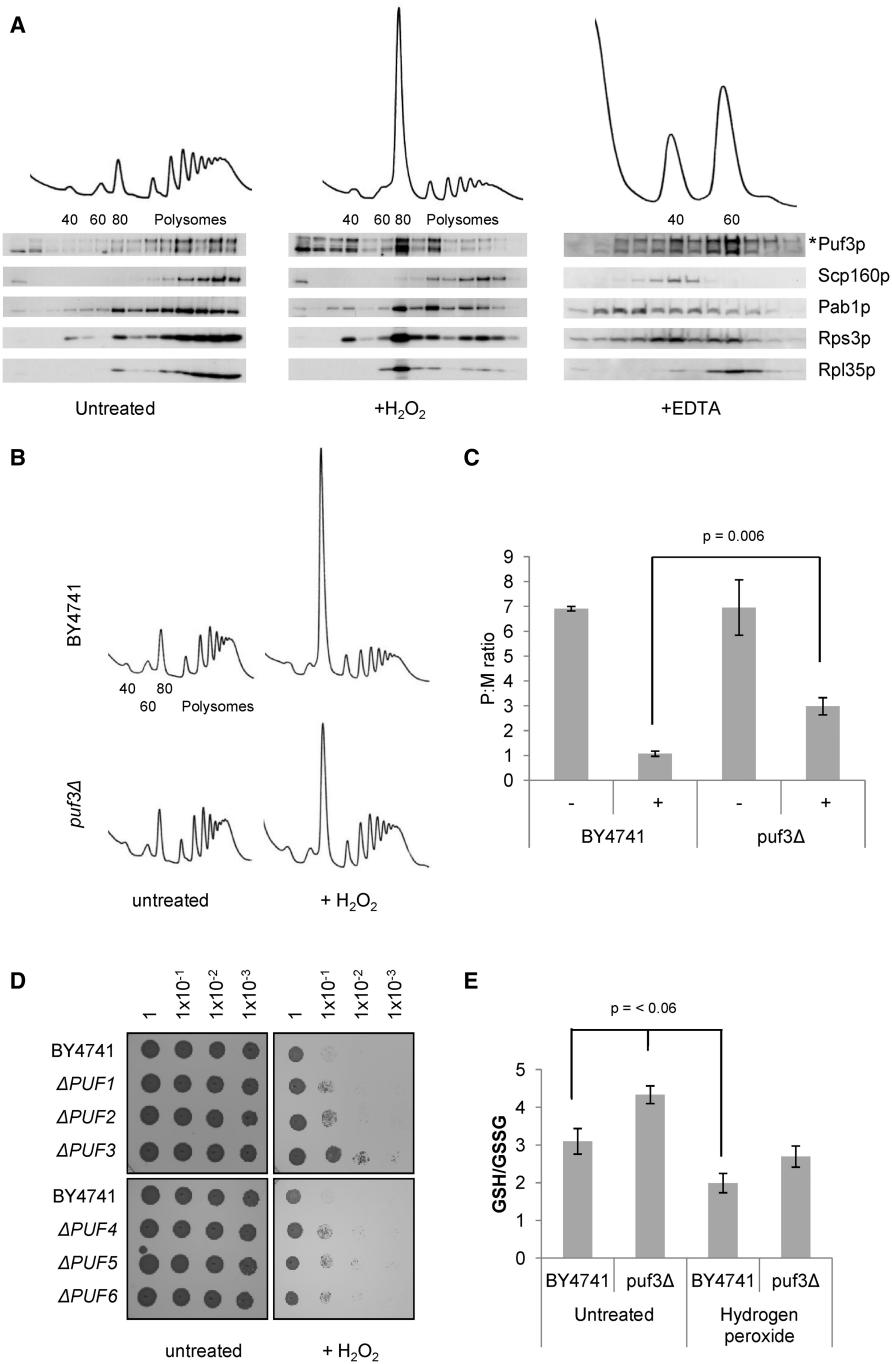
Translational properties of yeast wild-type and Puf3 mutants under oxidative stress. Polysome traces for the BY4741 PUF3-TAP strain with and without hydrogen peroxide treatment, and in the presence of EDTA. The 40S small ribosomal subunit, the 60S large ribosomal subunit, the 80S monosome and the polyribosomes are labelled. Fractions were analysed by immunoblotting using antibodies raised against the proteins specified adjacent to each panel with the exception of Puf3p-TAP that was detected using a PAP antibody. Asterisk indicated both bands represent Puf3p-TAP signal. Immunoblotting of Rps3 and Rpl35 are used as small ribosomal and large ribosomal subunit markers, respectively (**A**). Polysome traces of wild-type and *puf3Δ* strains (**B**). These polysomes profiles were performed in triplicate, the monosomal and polysomal peaks quantified using Image J and translational repression is shown as a mean ratio of polysomes:monosomes (**C**). Growth of a serial dilution of *puf1Δ*, *puf2Δ*, *puf3Δ*, *puf4Δ*, *puf5Δ* and *puf6Δ*, and wild-type (BY4741) strains under control conditions and 14 mM hydrogen peroxide concentrations. The OD_600_ of each dilution is indicated at the top of each column (**D**). Intracellular oxidation state described as a mean ratio of oxidized glutathione (GSH):reduced glutathione (glutathione disulfide—GSSG) of wild-type BY4741 and *puf3Δ* performed in triplicate (**E**). In all cases, error is shown as standard error of the mean.

The Random Forest analysis indicates that there is an association between Puf3p and transcripts that are translationally repressed in response to oxidative stress. We therefore studied the association of Puf3p with different fractions in a polysome gradient. Polysome extracts of a BY4741 Puf3p-TAP tagged strain were resolved on 15–50% sucrose gradients. Fractions were collected across the gradients and analysed by western blotting to assess whether Puf3p-TAP co-sediments with translating (poly)ribosomes. In addition, four other proteins were assessed in this manner: Pab1p, Scp160p (a control protein that is not associated with transcripts translationally repressed under oxidative stress) and two ribsosomal proteins, Rps3p and Rpl35p (Figure 6A).

Puf3p-TAP co-sediments in a sucrose gradient with the monosome and polysomes under unstressed conditions and this co-sedimentation is lost after hydrogen peroxide stress with the majority of Puf3p-TAP migrating with the 80S monosome and lighter ribosome-free fractions (Figure 6A). Scp160p also co-sediments with the polysomes but, unlike Puf3p-TAP, this co-sedimentation is only modestly affected by hydrogen peroxide. This is despite the general global shift of many transcripts into the monosomal fractions. The sedimentation of Pab1p is also affected by hydrogen peroxide but not to the same extent as Puf3p-TAP. Pab1p appears to follow the trend expected for the majority of the mRNA and the ribosomal subunits as they dissociate, a consequence of the general down-regulation of protein synthesis under stress. Thus Puf3p exhibits a more extreme migration shift out of polysomes following oxidative stress than Pab1p, even though they apparently share many mRNA targets (17,23).

As a control, polysome extracts were also treated with EDTA to dissociate the ribosome into its constituent 40S and 60S subunits (Figure 6A, third panel). Puf3p-TAP co-sediments with both the 40S and 60S ribosomal subunits and therefore remains associated after EDTA treatment. This is consistent with other recent studies that show evidence for an RNA-dependent interaction between Puf3p and other ribosomally associated proteins such as Pab1, eIF4E, eIF4G, as well as both small and large ribosomal subunits, corroborating our observed ribosome interaction as opposed to interaction with other large molecular weight complexes. It is not yet clear if the interaction of Puf3p with ribosomes is direct or mediated by other ribosome-bound factors (23).

To assess Puf3p’s effect on global translation after oxidative stress, polysome profiles were analysed from wild-type BY4741 and *puf3Δ* strains with and without hydrogen peroxide stress. When stressed with hydrogen peroxide, wild-type cells behave identically to the Puf3p-TAP strain and demonstrate a characteristic shift of the mRNA pool from polysome fractions to the 80S monosomal peak compared with the unstressed conditions, indicative of translational repression (Figure 6B). However, in the *puf3Δ* strain, this shift is much less pronounced, indicating that, in the absence of Puf3p, global translational repression is diminished following hydrogen peroxide treatment. When integrating the area under the respective traces to calculate polysomal:monsomal ratios for the four conditions, we note that the quantitative difference between the wild-type and *puf3Δ* strain is statistically significant with *P* < 0.006 (Figure 6C).

### *puf3Δ* strain exhibits increased resistance to hydrogen peroxide stress

To study whether these effects have any physiological relevance to yeast growth, the phenotypic response of the *puf3Δ* yeast strain was studied by measuring cell growth under high (14 mM) peroxide concentrations that are lethal to wild-type cells. For comparison, knockout strains for each of the five other PUF gene-deleted strains were also examined. Each of the PUF knockout strains showed a modest increased robustness in terms of growth on hydrogen peroxide plates relative to the wild type (Figure 6D). This effect is most prominent for the *puf3Δ* mutant, which has the most robust growth under oxidative stress relative to the other PUF-protein knockout and wild-type strains. This altered growth phenotype and altered translational response (Figure 6 B and C) indicates that association of Puf3p with its targets plays an important role in the normal response to oxidative stress.

### *puf3Δ* strain has an altered glutathione redox state

As both growth and translation in the *puf3Δ* mutant are more resistant to hydrogen peroxide, we also considered whether the internal oxidative balance of the cytoplasm is altered in the mutant. To assess the internal oxidative state of these strains, the levels of both reduced glutathione (GSH) and oxidized glutathione (GSSG) were measured. The ratio of reduced to oxidized glutathione is significantly higher in the cytoplasm of the *puf3Δ* mutant compared with the wild-type strain, indicative of a more reduced cellular environment (Figure 6E). As expected, this ratio drops under oxidative stress in both yeast backgrounds. However, in *puf3*Δ cells, the increased ratio of GSSG is not significantly different from the ratio in unstressed wild-type cells. As with the previous results obtained, this data points to altered metabolism in *puf3*Δ cells so that they do not down-regulate translation and cell growth as wild type cells do.

### Puf3p continues to bind to its target mRNAs after oxidative stress

**Figure 7.**
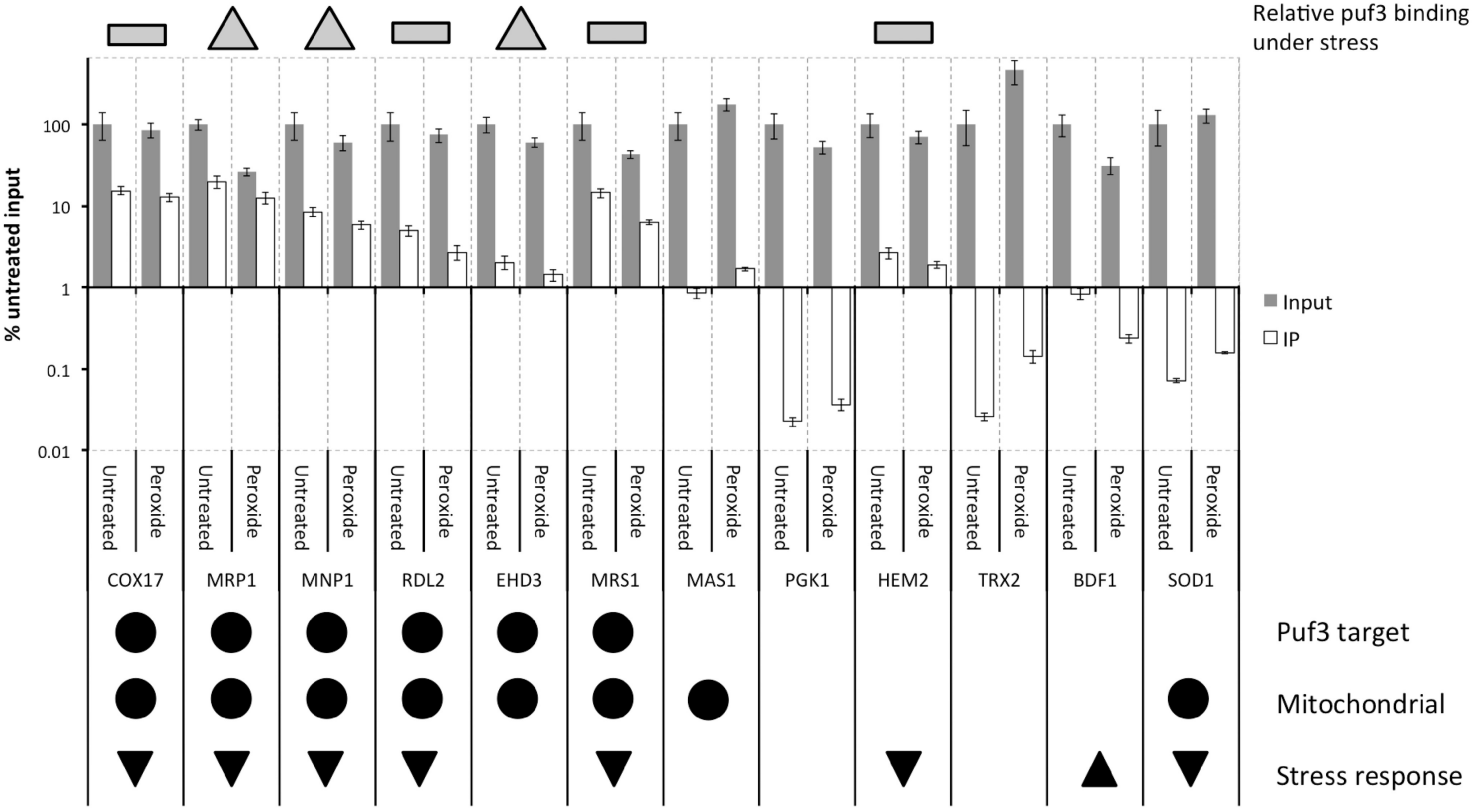
Transcript levels and Puf3p binding of selected transcripts in WT and hydrogen peroxide stressed conditions. Transcript and Puf3p-TAP RNA-IP levels for six Puf3p targets and six control mRNAs in untreated (grey) and peroxide-treated (white) conditions. RNA was quantified using qPCR. Data are shown as a mean of biological triplicates. Error is shown as standard error of the mean. For those transcripts observed to be bound by Puf3 (minimum 1% of WT level in both stressed/unstressed), the relative change in level after stress is indicated as either an increase (greater than the measured standard error) by a grey triangle, or no change (grey rectangle). Properties of each mRNA are also shown below; known Puf3 targets according to (17) and those annotated as mitochondrial by SGD (58) are indicated with black filled circles below the PCR data. Also shown is the translational response of each mRNA in the Shenton *et al.* data set (30): translation increased (black arrow pointing up), decreased (black arrow pointing down), otherwise, no change in response to peroxide.

The Random Forest analysis highlights the link between Puf3p target mRNAs and enhanced translational repression following a short-term oxidative stress (Figures 3 and 4), while the polysome analysis shows the dramatic movement of Puf3p out of polysomes following stress (Figure 6A). To determine if Puf3p-target transcripts remain associated with Puf3p following hydrogen peroxide treatment, we carried out qRT-PCR experiments on a representative set of 12 transcripts. Whole-cell extracts were generated from Puf3p-TAP strains that had been treated with hydrogen peroxide or left unstressed. Puf3p-TAP immunoprecipitations were performed on these extracts to isolate associated mRNA. This mRNA, in addition to samples of total RNA isolated from the IP inputs, was then converted to cDNA and quantified via qPCR. To account for different mRNA concentrations on a transcript-by-transcript level, all data were normalized as a percentage of the untreated input sample. These data therefore show the changes in the levels of individual mRNAs in response to oxidative stress as well as the relative fraction bound by Puf3p (Figure 7). Transcripts were selected to represent a variety of properties: known Puf3p targets from previous RIP-ChIP (17), mitochondrially expressed according to SGD (58) and the translational response of each gene in response to oxidative stress (30). These partitioned into six previously characterized Puf3p targets and six non-targets.

As expected, the six well-characterized Puf3p target mRNAs are enriched in the Puf3p-TAP immunoprecipitations (*COX17*, *MRP1*, *MNP1*, *RDL2*, *EHD3* and *MRS1*) and binding of these targets by Puf3p is generally not significantly reduced after hydrogen peroxide stress (Figure 7). This is particularly clear for *COX17* and *MRP1*. For *MRP1*, *MNP1* and *EHD3*, the relative level of Puf3p binding increases after stress as indicated by the grey up-arrows, while it remains at the same relative level for *COX17*, *RDL2* and *MRS1*. *MRP1* and *MRS1* are the only Puf3p targets that are significantly reduced by hydrogen peroxide treatment at the transcript level; however, in both cases, this is not reflected in a reduction in Puf3p binding. This is entirely consistent with Puf3p’s role as a translational repressor, which continues to function despite transcriptional down-regulation under stress. We did not observe any significant binding of Puf3p as expected in any of the other transcripts, with the notable exception of *HEM2*. We now have data to suggest that *HEM2* is a novel Puf3p-target RNA from a high throughout study (data not shown), bound either directly by Puf3p or indirectly by a protein–protein interaction maintained under our experimental conditions [c.f. (23)].

In summation, we observe Puf3p moves out of polysomes in response to oxidative stress, but continues to bind its target mRNAs, thereby removing them from actively translating ribosomes. Collectively, these data indicate that translation of these mRNAs is repressed in response to oxidative stress.

## DISCUSSION

Oxidative stress leads to the translational regulation of hundreds of mRNAs as revealed by polysomal profile analysis in combination with microarray analysis (30). It has previously been shown that under oxidative stress conditions, *S. cerevisiae* responds through Gcn2p-mediated inhibition of translation initiation (30). This would be expected to result in congruence between changes in transcript level and changes in polysome association in an effect known as potentiation. The lack of observed congruence therefore indicates that many transcripts may be subject to additional specific translational controls, either by activation or ‘super’-repression beyond the global translational repression that is observed for the majority of genes in response to oxidative stress. This in turn suggests that there are characteristics specific to these mRNAs that may dictate translational control mechanisms.

Our meta-analyses of translatome data sets confirm this and reveal a significant co-association between Puf3p targets and translational down-regulation of transcripts under oxidative stress, consistent with studies indicating Puf3p role as a repressor of translation (61,72). This general feature of the PUF proteins to act as a repressor is widely reported in eukaryotes; for example, the homologous protein *Pumilio* can repress translation in *D. melanogaster* through interaction with the eIFs (73); however, this is the first study, to our knowledge, to show that control is exerted in response to stress.

Our data are also consistent with previous observations of Puf3p translational control in yeast linked to mitochondrial function (67), targeting many nuclear encoded genes involved in mitochondrial protein synthesis. The mitochondria is the main cellular production plant of ROS (70), and disruption of the respiratory chain can lead to an overproduction of ROS leading to cellular ageing and death. As the respiratory chain is itself susceptible to oxidative stress, this may induce a positive feedback loop, to the detriment of the cell. Given that Puf3p binds to mRNAs of proteins destined for the mitochondria as well as proteins associated with the mitochondria (71), it makes physiological sense for cells sensing elevated oxygen radicals to possess the ability to down-regulate the synthesis of proteins whose function will potentially contribute to further increases in toxic species. Thus we propose a stress-activated Puf3p-mediated translational repression contributing to a mechanism to temporarily limit synthesis of new mitochondrial proteins as cells adapt to the stress.

However, such correlations do not necessarily imply a direct causal relationship between Puf3p and translational regulation following oxidative stress; further experimental validation that Puf3p is acting as a specific regulator of translation *in vivo* is needed. The analysis presented in Figure 6A is consistent with this hypothesis, where we observe that epitope-tagged Puf3p is more highly associated with transcripts in the monosomal fraction following oxidative stress, contrary to other translational markers, indicating a shift away from actively translating polysomes. This effect is not observed for other genes we considered, including Scp160p an RNA-binding protein known to be involved in translational control (74).

Further, when studying the polysome traces of the *puf3Δ* knockout strain, we observed that the characteristic general downshift of mRNA from the polysome peak to the lighter monosomal fractions is significantly reduced following oxidative stress (Figure 6B and C). This is consistent with Puf3p playing a repressive role and helping reduce the rate of translation following stress, since its absence permits more active translation. This is complimented by the Cell growth studies (Figure 6D), which reveal that the *puf3*Δ strain has an increased robustness to oxidative stress, and by increased GSH:GSSG ratio (Figure 6E). Although these results point to Puf3p’s role in the normal response to oxidative stress, it is unclear mechanistically how loss of Puf3p can improve cell survival in response to oxidative stress. It is known that Puf3p has a role in localization of specific transcripts to mitochondria (75) and that mitochondria from *puf3*Δ cells possess increased abundance of proteins required for oxidative phosphorylation and have enhanced oxygen production (67). These observations complement ours and suggest that the altered mitochondrial function in *puf3*Δ cells may well contribute to their enhanced survival. Identifying which Puf3p targets are directly responsible is not straightforward and is beyond the scope of this study, particularly given, the multiple functional roles ascribed to Puf3p and potential fates for its targets in response to peroxide.

These analyses are consistent with Puf3p’s role as a repressor of translation. This repression appears to be exacerbated under oxidative stress, and may form a crucial regulatory mechanism in the cell’s response to oxidative stress. The PUF proteins are known regulators of translation, and in some cases their precise modes of action have been characterized; for example, the homologous protein *Pumilio* can repress translation in *D. melanogaster* through interaction with the eIFs (73). These data are also consistent with previous observations of Puf3p translational control in yeast (67). The mitochondrion is the main cellular production plant of ROS (70), and disruption of the respiratory chain can lead to an overproduction of ROS, leading to cellular ageing and death. As the respiratory chain is itself susceptible to oxidative stress, this may induce a positive feedback loop, to the detriment of the cell. Puf3p interacts with proteins associated with the mitochondria (71), and hence, an inhibitory role in response to oxidative stress is perhaps an unsurprising discovery.

As already noted, it is difficult to directly determine the cause of the increased robustness of the *puf3*Δ strain to peroxide. One possible explanation is via the up-regulation of genes required to detoxify or protect against ROS, although further work would be required to confirm this. For example, Puf3p binds the transcripts of the redox enzyme Grx5, which is known to increase robustness of the cell to oxidative stress (76). In the short term, reduction in the inhibition of translation of such enzymes may be responsible for the increased robustness observed in the *puf3Δ* strains. An alternative explanation stems from Puf3p’s multipurpose functionality, since it can affect transcript levels through increased degradation in addition to regulating translation. Puf3p is known to mediate transport of mRNA to the mitochondria (71); inhibition of this process may provide a different explanation for the increased robustness in the knockout strain under oxidative stress.

## CONCLUSIONS

These data presented here show the power of computational biology to uncover novel findings and generate experimentally testable hypotheses from postgenomic data sets concerning the regulation of gene expression. This is particularly informative when applied to organisms with the rich resources of yeast, leading to an iterative cycle of informatics and experimental science. Interestingly, a similar approach to ours using a Random Forest to mine features associated with discordance between transcript levels and protein levels, did not identify Puf3p as a significant factor (77), although Puf3 targets were not used explicitly in the model, though those of several other RBPs were. However, both studies highlight the power of machine learning analysis to mine existing postgenomic data sets for significant posttranscriptional control features, leading to hypotheses that can be experimentally validated.

Our study highlights the importance of translational control as a means to regulate mitochondrial proteins in response to oxidative stress. Peroxide stress has been implicated in numerous human diseases including Alzheimer’s, diabetes and Parkinson’s disease (78) and yeast remains a key model system to investigate the fundamental molecular events in the study of human mitochondrial disorders (79). Yeast can survive through respiration or fermentation, and knockouts of key mitochondrial genes can still be studied if prepared on the right media, highlighting the utility of *S. cerevisiae* to study translational control.

Our results indicate a likely role Puf3p in the control of translation under oxidative stress conditions, linking its role in repression of protein synthesis to mitochondrially active genes, and acting as a starting point for a more rigorous experimental analysis to determine the precise mode of action.

## SUPPLEMENTARY DATA

Supplementary Data are available at NAR Online.

## FUNDING

Biotechnology and Biological Research Council (UK) grant [BBG012571]. Funding for open access charge: Biotechnology and Biological Sciences Research Council (BBSRC) [BBG012571].

*Conflict of interest statement*. None declared.

## Supplementary Material

Supplementary Data
