# Supplementary material for: Puf3p induces translational repression of genes linked to oxidative stress
Source: Nucleic Acids Res. 2013 Oct 25;42(2):1026–41. doi: 10.1093/nar/gkt948 (PMC3902938; doi:10.1093/nar/gkt948)
Supplement: Supplementary Data [file supp_42_2_1026__index.html]

Puf3p induces translational repression of genes linked to oxidative stress — Puf3p induces translational repression of genes linked to oxidative stress — Supplementary Data 

# Puf3p induces translational repression of genes linked to oxidative stress

## Supplementary Data

files

**Files in this Data Supplement:**

- Supplementary Data - xls file
- Supplementary Data - docx file
