# Supplementary material for: Puf3p induces translational repression of genes linked to oxidative stress
Source: Nucleic Acids Res. 2013 Oct 25;42(2):1026–41. doi: 10.1093/nar/gkt948 (PMC3902938; doi:10.1093/nar/gkt948)
Supplement: Supplementary Data [file supp_gkt948_nar-02298-a-2013-File003.docx]

**Supplementary Table 2: qPCR primer sequences**

| Primer | Sequence |
| --- | --- |
| Bdf1 F | CGGGTTTTTCTTGTCCCTTA |
| Bdf1 R | CGACCGCCTTTTAGCTTGT |
| Cox17 F | AAAGGAGGAGCGGGATACAT |
| Cox17 R | GCCATAACCCTTCATGCACT |
| Ehd3 F | GCCTGTGTACGGGAATCCT |
| Ehd3 R | TTAGACGACAATTGGGCACAT |
| Hem2 F | CCGTGATGCTGCTTGTTCAG |
| Hem2 R | CCTTTCCAACGCCCTACGTG |
| Mas1 F | AGAAGGCGTGTCATGGTCTG |
| Mas1 R | GGGAGTTTGTACCTGTGCCA |
| Mnp1 F | TCGTTCGACACGAAGACTAAGG |
| Mnp1 R | TTGGTGCAGCTTCAACGAAT |
| Mrp1 F | TGGGTCTTTGATGGAGTT |
| Mrp1 R | TTGCGACTAACCACGTAA |
| Mrs1 F | CTGCGATCCGCCTTCGATAA |
| Mrs1 R | TAGGCCCGTTGTCGAGGATA |
| Pgk1 F | TTGCCAGGTGTTGCTTTCTT |
| Pgk1 R | TAGCGTAAAGGATGGGGAAA |
| Rdl2 F | GCTCTTGGATTGCCCGAAAAG |
| Rdl2 R | TTGGCTCTTACTCCTTTCGCA |
| Sod1 F | CGAGCCAACCACTGTCTCTT |
| Sod1 R | AGACACAACCATTGGTGGCA |
| Trx2 F | TCCGCTTCTGAATACGACAGT |
| Trx2 R | CATTTTACATGGCCCACACCA |
